# Recent Advances in Fiber Bragg Grating Sensing

**DOI:** 10.3390/s24020532

**Published:** 2024-01-15

**Authors:** Antreas Theodosiou

**Affiliations:** Photonics Research Center (PRC), Lumoscribe Ltd., Anthipoloxagou Georgiou M Savva 12, 8201 Paphos, Cyprus; theodosiou.antreas@lumoscribe.com

## 1. Introduction

In the vast realm of optical fiber sensing, where precision and innovation converge, Fiber Bragg Gratings (FBGs) stand as luminaries, casting their influence across myriad applications [1]. These microscopic structures within optical fibers have become the bedrock of cutting-edge sensor technologies, revolutionizing the landscape of data acquisition and measurement. As we embark on this editorial review, our focus is unwaveringly set on the recent research advancements in FBGs and their applications in optical fiber sensors, offering a panoramic view of the strides taken in this dynamic field.

The journey begins with the fundamental understanding of Fiber Bragg Gratings—a triumph of ingenuity where periodic variations in the refractive index within an optical fiber create a unique spectral pattern [2,3]. This simple yet powerful concept has become a cornerstone in the development of optical sensors, providing an elegant solution for measuring various physical parameters with unparalleled precision.

Over the years, the scientific community has relentlessly pushed the boundaries of FBG technology, leading to a cascade of breakthroughs in sensor design, fabrication techniques, and applications [4,5,6]. Our exploration will delve into the innovative strides made in the fabrication of FBGs, from traditional UV-induced gratings [7] to advanced techniques like femtosecond laser inscription [8,9], offering readers a glimpse into the evolution of the fabrication methodologies that underpin the success of optical fiber sensors.

As we traverse through the editorial landscape, the spotlight will shine on the diverse range of applications in which FBGs have demonstrated their prowess. From structural health monitoring and environmental sensing to biomedical applications and beyond [10,11], the versatility of FBGs continues to astound researchers and engineers alike. We will navigate through recent case studies and exemplary research projects, shedding light on how FBGs have become indispensable tools in addressing contemporary challenges across various domains.

Furthermore, this review will cast a forward-looking gaze into the future of FBGs and optical fiber sensors, exploring emerging trends and potential avenues for further exploration. The integration of FBGs with other technologies, such as artificial intelligence and machine learning, promises to elevate the capabilities of optical sensors, paving the way for unprecedented levels of data accuracy and real-time monitoring.

In conclusion, this editorial review aspires to be a beacon guiding readers through the intricate web of advancements in Fiber Bragg Gratings and optical fiber sensor technologies. With each page turned, we invite you to join us in unraveling the threads of innovation that have woven a tapestry of progress in this captivating field, igniting a vision of a future where optical fiber sensors redefine the limits of what we can measure and understand.

## 2. An Overview of the Published Articles

The article from Arnaldo et al. (contribution 1) focuses on the development and characterization of Fiber Bragg Grating (FBG) sensors coated with ultraviolet (UV)-curable resins, specifically NOA 68 and NOA 88. The UV-curable resins were applied after FBG inscription and cured with a UV lamp. The study investigates the impact of these coatings on temperature, moisture absorption, and strain responses. Tensile tests were conducted to understand the influence of resin coating on the optical fiber’s mechanical properties.

The results indicate that moisture absorption was negligible for both coatings during a 50 min test in distillated water. The temperature responses of the coated FBGs were higher compared to uncoated ones, which was attributed to the thermal expansion of the coatings. In strain tests, both the coated and uncoated samples showed similar sensitivities, but the coated samples exhibited a larger strain range.

The stress–strain curves revealed that the Young’s modulus of the coated samples was one order of magnitude lower than that of uncoated silica fiber, with the NOA 68 coating showing the lowest modulus (3.84 GPa). This suggests the potential for highly sensitive pressure and force sensors. The proposed method is deemed simple, low-cost, and provides robust fiber coating. Additionally, the in situ application of coatings is highlighted as a significant advantage over other coating approaches for optical fibers.

In conclusion, the paper presents a viable alternative for enhancing FBG sensors in silica fibers for mechanical sensing by using UV-curable resins as protective coatings. The coated FBGs demonstrate improved temperature sensitivity and a broader strain range, making them suitable for various sensing applications. The research suggests that UV-curable resins offer a low-cost, highly customizable, and straightforward method for FBG coating, showcasing potential advantages over uncoated fibers in sensing applications. Future work is proposed to explore additional resins and investigate the trade-offs between coating layer thickness, material, and sensor performance.

Violakis et al. (contribution 2) explored the use of optical-fiber-based acoustic emission (AE) detection sensors, specifically Fiber Bragg Grating (FBG) sensors, for structural health monitoring or medical diagnosis. The study compares FBGs inscribed in optical fibers with diameters of 50, 80, and 125 μm, with a focus on the advantages of smaller-diameter fibers. The edge filter detection method is employed to capture reproducible acoustic waves, and the acquired signals are analyzed using fast Fourier and wavelet decompositions.

The results reveal that, despite the 50 μm diameter optical fiber being more prone to noise due to non-standard splicing methods, it exhibits a higher sensitivity and a broader dynamic range than its larger-diameter counterparts. The research highlights the advantages of using smaller-diameter optical fibers for AE detection, providing superior sensitivity and performance across the entire ultrasonic range.

In its conclusion, the study emphasizes the significant benefits of smaller optical fiber diameters (50 μm) for ultrasonic detection, showcasing a higher sensitivity and a dynamic range. The findings are supported by both fast Fourier transform and wavelet decomposition analyses, with the latter offering additional advantages in terms of sensitivity and dynamic range mapping over the full frequency response range. The paper suggests potential improvements in AE detection sensitivity for the 50 μm diameter optical fiber by optimizing the connection to standard-diameter fibers, reducing mode field diameter mismatch losses and enhancing the optical fiber holder design. Furthermore, the transition from silica optical fibers to polymer optical fibers is proposed as a means to achieve even higher AE detection sensitivity. Overall, the study underscores the advantages of using smaller-diameter optical fibers in AE detection and provides insights into potential avenues for further performance enhancements.

Goossens et al. (contribution 3) investigated the impact of different Fiber Bragg Grating (FBG) lengths on the detection of ultrasonic guided waves (UGWs) for non-destructive inspection (NDI) purposes. The study aims to experimentally validate the effect of the ratio between the UGW wavelength (λ_UGW_) and the FBG length (L) on the FBG’s spectral response during the passage of a UGW. The analysis involves a full spectral reconstruction of the Bragg peaks for different FBG lengths, with a focus on understanding how UGW-induced strain affects the Bragg peak.

Their experimental findings confirm the importance of the UGW wavelength to FBG length ratio (λ_UGW_/L) and its impact on the Bragg peak. For λ_UGW_/L values approaching unity, the strain on the FBG becomes non-uniform, leading to Bragg peak broadening. However, the study reveals that, even when peak broadening dominates, the shape of the first wave packet of the UGW signal remains consistent, allowing for successful detection through edge-filtering interrogation.

The conclusions emphasize that the commonly assumed requirement of λ_UGW_/L ≈ 1 may not be strictly necessary. The research shows that, despite the dominance of Bragg peak broadening with increased FBG length, UGW signals can still be effectively acquired through edge-filtering. Additionally, longer FBG sensors offer higher sensitivity, leading to larger signal amplitudes and potentially improved measurement quality. Therefore, the study suggests that, contrary to previous assumptions, fulfilling the specific condition of λ_UGW_/L ≈ 1 is not strictly mandatory for successful UGW detection with FBGs, and longer FBG sensors might even be preferred for enhanced sensitivity and measurement quality.

Theodosiou et al. (contribution 4) introduced a monolithic fiber laser operating in the short wavelength infrared, specifically designed for CO_2_ gas sensing applications. The laser design is based on the direct inscription of a monolithic Fabry–Perot cavity in a thulium-doped optical fiber, employing the femtosecond laser plane-by-plane inscription method to create the cavity mirrors. Two wavelength-identical fiber Bragg gratings (FBGs) with high and low reflectivity are used for cavity construction.

The paper details the characterization of a laser cavity with a single high reflectivity FBG, followed by the development of three fully monolithic cavities. The lasers are designed for continuous wave operation at 1950 nm and evaluated based on power output, slope efficiency, stability, and effective resonator length. The monolithic laser cavities exhibit promising results, with an improvement exceeding 12% in slope efficiency compared to previous work using the same thulium-doped fiber but with FBGs inscribed in silica-passive fiber.

In conclusion, the study successfully demonstrates a fully monolithic fiber laser applicable to CO_2_ gas sensing in the short wavelength infrared region. The characterization of the laser cavities, including the effective length and power slope efficiency, is performed systematically. The reproducibility of the method is verified through the development of three monolithic fiber laser cavities, each incorporating a short, broad bandwidth, and low reflectivity FBG. The results showcase a high degree of reproducibility, emphasizing the potential of the proposed monolithic fiber laser for practical applications in gas sensing.

Rodriguez-Garrido et al. (contribution 5) focused on employing femtosecond-laser-inscribed Fiber Bragg Gratings (FsFBGs) for monitoring the high-temperature surface distribution (HTSD) within concentrating solar power (CSP) plant solar receivers. The fiber-optic sensor system, consisting of 12 FsFBGs distributed over 0.4 m^2^, enables the creation of a temperature map through two-dimensional interpolation. The study assesses the FsFBG performance in harsh environments, considering calibration functions, spectral intervals, and fabrication tolerances. Their results indicate the reliability of this technique, with temperature measurements successfully recorded up to 566 °C.

The application of the fiber-optic sensor system was tested on a central receiver prototype in a CSP tower facility, demonstrating its effectiveness in capturing HTSD under various solar irradiance distributions. While the concept proved successful, the research identifies critical aspects for improvement in the sensor design.

In conclusion, the proposed fiber-optic sensor system, utilizing FsFBGs, was validated for monitoring temperature surface distribution in CSP plant central receivers. The system’s viability was demonstrated in a specially designed prototype, which successfully recorded characteristic temperature maps at elevated temperatures. The study emphasizes the importance of FsFBG design and performance analysis, considering wavelength peak tolerances, spectral intervals, and calibration functions. Future work will explore the application of regenerated FBGs for higher temperature measurements, increasing the number of sensor elements for improved spatial resolution and analyzing calibration functions for FsFBG and regenerated FBG systems. This research highlights the promising potential of high-temperature monitoring with FsFBGs in diverse industrial applications, including aerospace, nuclear plants, oil and gas exploration, and advanced robotics in harsh environments.

Shin et al. (contribution 6) focused on the development of a Fiber Bragg Grating (FBG) force sensor system for cardiac catheterization applications, leveraging the advantages of FBG sensors, such as their being lightweight, low-cost, and free from electromagnetic interference. Two different flexure structures were designed and simulated to determine their sensitivity and durability using ANSYS software. The selected helical structure, found to be more sensitive, was combined with three FBGs and an interrogator to create a tri-axial FBG force sensor at the catheter tip.

The FBG force sensor system was calibrated by measuring the change in wavelength with force, achieving a high resolution of 0.01 N within the 0.1–0.5 N range. The LabVIEW program was utilized to generate a calibration curve, enabling the real-time measurement of unknown force values during the catheter ablation procedure. The study also compared the sensitivity of helical and perforated flexure structures and demonstrated the repeatability and stability of the FBG force sensor.

In conclusion, the developed FBG force sensor system offers advantages such as high repeatability and stability, making it suitable for cardiac catheterization procedures where interference with electronic devices in the operating room needs to be avoided. The helical flexure structure, combined with FBGs, proves to be an effective and sensitive configuration for real-time force sensing. The system’s potential application in cardiac catheterization is highlighted, emphasizing its reliability for physicians in medical procedures.

Eiserman et al. (contribution 7) focused on the metrological investigation of Fiber Bragg Gratings (FBG) inscribed in single crystalline multimode sapphire fibers (S-FBG) for high-temperature applications up to 1900 °C. A hybrid optical temperature sensor, incorporating S-FBG, thermal radiation, and a conventional thermocouple, was developed and tested in an industrial silicon manufacturing process. The study aimed to analyze the achievable temperature uncertainties and enhance the signal processing of the reflected S-FBG spectrum.

The results show that the hybrid sensor, combining S-FBG with thermal radiation and a calibrated thermocouple, performed robustly in harsh environments. The S-FBG sensor demonstrated stability against mechanically caused mode variations and aging effects caused by high-temperature processes. Calibration with temperature-fixed points enabled accurate measurements up to 1600 °C, with the S-FBG and thermocouple measurements agreeing within their combined uncertainties.

The conclusions highlight the successful metrological characterization of the high-temperature hybrid sensor, emphasizing its application in monitoring industrial processes in challenging conditions. The study proposes strategies to further reduce the uncertainty of S-FBG calibration, making them a promising choice for high-temperature measurements, with potential competitiveness with thermocouples while benefiting from fiber-based sensing advantages.

Nan et al. (contribution 8) investigated the characteristics of Fiber Bragg Gratings (FBG) in cyclic transparent fluoropolymer (CYTOP) optical fiber, focusing on the FBG response to temperature, humidity, and strain. Two uniform FBGs were inscribed separately in CYTOP fiber with and without over-clad. A three-variable two-level factorial experimental method was employed to study the sensitivities and cross-sensitivities of these parameters. The interactions among temperature, humidity, and strain were analyzed, and the parameter sensitivities were estimated.

The results reveal similar temperature and strain sensitivities for both FBGs, while a significant cross-sensitivity between humidity and temperature is present only in the FBG inscribed in CYTOP fiber with over-clad. The study highlights the hydrophilicity of the over-clad material, impacting the temperature sensitivity. The research provides insights into the complex interactions among environmental parameters in CYTOP-FBGs, offering valuable information for applications requiring accurate sensing in such fibers.

Liang et al. (contribution 9) introduced a novel three-dimensional stress-monitoring method for surrounding rocks in roadways using Fiber Bragg Grating (FBG) sensing technology. A cube-shaped three-dimensional stress fiber grating sensor is developed based on this method. The study validates the sensor’s feasibility through theoretical analysis, simulation, and experimentation, showcasing its application in underground engineering safety monitoring, particularly in coal mines and tunnels.

The sensor’s performance is tested through a uniaxial compression experiment, demonstrating a high sensitivity to X, Y, and Z axis stress with a relative error of less than 4%. The sensor exhibits excellent linearity and repeatability. The research holds significance for understanding the distribution and evolution of surrounding rock stress fields, preventing coal and rock dynamic disasters, optimizing roadway support parameters, assessing risks in working face areas, and enhancing mine safety production.

In conclusion, the paper presents the design principles, numerical relationships, and sensitivity formulas of the three-dimensional stress sensor. The ANSYS Workbench numerical simulation validates the sensor’s performance under uniaxial compression, ensuring accurate stress measurements. The experimental results indicate high linearity, repeatability, and minimal relative error, demonstrating the sensor’s potential for practical applications. The paper suggests future research directions, including temperature compensation, FBG protection, and the development of three-dimensional stress-monitoring software for enhanced data processing.

Lin et al. (contribution 10) introduced a novel algorithm for the automatic demodulation of surrounding refractive index (SRI) variations in a Tilted Fiber Bragg Grating-Surface Plasmon Resonance (TFBG-SPR) sensor probe. The algorithm involves three key steps: peak identification and cladding mode selection, the determination of the most sensitive cladding mode, and efficiency and sensitivity enhancement through cladding mode fitting techniques. The paper focuses on reducing the spectral scanning time and improving SRI sensitivity.

The algorithm is validated by preparing refractive indices with a tiny index step using a glucose aqueous solution. The study demonstrates the automatic selection of the most sensitive cladding mode, achieving a sensitivity of −6887 dB/RIU (refractive index unit) with a scanning time of 15.77 s. The researchers explore the trade-off between scanning speed and sensitivity by varying the wavelength resolution, optimizing the algorithm for practical application.

In conclusion, the proposed algorithm streamlines the calibration and measurement of SRI in TFBG-SPR sensor probes. The research highlights the potential for automatic calibration and ease of application, setting the stage for integration into commercial devices in the future. The improvements in efficiency and sensitivity contribute to the advancement of SPR-based sensing technologies.

Liang et al. (contribution 11) addressed the challenges of existing Fiber Bragg Grating Force-Measuring Bolts (FBG-FMB) in mine roadway support and introduced a novel solution—the desensitized FBG-FMB. The conventional FBG-FMB faced issues such as fiber breakage and sensing ineffectiveness during elongation, leading to a mismatch between FBG elongation and the bolt’s strain. To address this, the study establishes a FBG strain desensitization sensing model and designs a spring-desensitized FBG sensor.

The feasibility and reliability of the desensitized FBG-FMB are verified through mechanical analysis, pull-out experiments, and field applications in a coal mine roadway. The new sensor resolves the problem of uncoordinated strain between the grating and the bolt, allowing for early warnings of bolt failure and for collecting essential data for optimizing roadway support parameters. The axial stress distribution along the bolt is observed in a field test, showcasing the reliability and applicability of the force-measuring bolt.

However, the study acknowledges its potential limitations, such as the influence of ambient temperature and the need for improvements in temperature compensation. The desensitized FBG-FMB also lacks a supporting data processing system, emphasizing the opportunity for future work in developing a comprehensive solution for visualizing the bolt state. Overall, the desensitized FBG-FMB proves to be a promising technology with significant application prospects in various underground engineering fields beyond coal mine roadway support.

Song et al. (contribution 12) addressed the critical need for the accurate perception of the straightness of a scraper conveyor in coal mines, crucial for the construction of intelligent working faces. A precision compensation model is proposed, focusing on the rotation error angle, to enhance the accuracy of the Fiber Bragg Grating (FBG) curvature sensor of the scraper conveyor. The correctness of the model is rigorously verified through theoretical analysis, numerical simulation, and experiments. The paper emphasizes the engineering application value and broad prospects of the compensation model in various contexts, including coal mine underground conveyors, submarine pipelines, and ground pipelines.

The compensation model is designed to address low sensing accuracy, mainly attributed to the rotation error angle. The model is validated through numerical simulations, with the curvature radius calculated according to the compensation model showing a relative error within 0.9% for each discrete point. The experiments further confirm the correctness of the compensation model, with the relative error of the curvature radius after fitting within 3% and deduced from each discrete point within 6%. The study concludes that the accuracy compensation model meets the requirements for the straightness perception accuracy of the scraper conveyor.

While the paper highlights the significance of the proposed model, it acknowledges its certain limitations, such as the lack of a more applicable rotation error angle measurement instrument and the complexity of incorporating a rotation angle sensor with the FBG curvature sensor. The influence of torsion on long-distance curvature detection is also recognized, paving the way for future research. Additionally, the paper does not cover temperature compensation design, emphasizing the need for further exploration in unfavorable conditions where temperature variations are significant.

## 3. Conclusions

In conclusion, this comprehensive review paper provides a panoramic view of the recent advancements in Fiber Bragg Gratings (FBGs) and their diverse applications in optical fiber sensors. The journey through the intricate landscape of FBG technology reveals its foundational role in the evolution of sensor technologies, offering elegant solutions for measuring various physical parameters with unparalleled precision.

The exploration of FBG fabrication techniques showcases the relentless pursuit of innovation within the scientific community, from traditional UV-induced gratings to cutting-edge methods like femtosecond laser inscription. These advancements underscore the continuous efforts to enhance the sensitivity, versatility, and applicability of FBGs in optical fiber sensors.

The spotlight on diverse applications highlights the remarkable versatility of FBGs, ranging from structural health monitoring and environmental sensing to biomedical applications and beyond. Each research contribution discussed in the review paper provides valuable insights into the specific advantages and challenges associated with FBG applications in various domains, offering a rich tapestry of possibilities.

Looking forward, the review casts a gaze into the future of FBGs and optical fiber sensors, exploring emerging trends and potential avenues for further exploration. The integration of FBGs with technologies like artificial intelligence and machine learning promises to elevate the capabilities of optical sensors, opening doors to unprecedented levels of data accuracy and real-time monitoring.

In essence, this editorial review serves as a beacon guiding readers through the dynamic and evolving field of Fiber Bragg Gratings and optical fiber sensor technologies. With each contribution, the paper unravels the threads of innovation that have woven a tapestry of progress, igniting a vision of a future where optical fiber sensors redefine the limits of what we can measure and understand. As we turn the pages of this review, we are invited to join the journey of discovery, where FBGs continue to illuminate new paths in sensing technology.
